# Iloprost Concentration‐Dependently Attenuates Platelet Function and Apoptosis by Elevating PKA Activity

**DOI:** 10.1111/jcmm.70403

**Published:** 2025-02-10

**Authors:** Xuexiang Wang, Shuang Chen, Jun Wan, Chunliang Liu, Yan Yan, Muhammad Shoaib Khan, Ziyu Zhao, Kang Sun, Renping Hu, Mengnan Yang, Yue Xia, Kesheng Dai

**Affiliations:** ^1^ Cyrus Tang Medical Institute, Suzhou Medical College, Jiangsu Institute of Hematology, the First Affiliated Hospital and Collaborative Innovation Center of Hematology, State Key Laboratory of Radiation Medicine and Protection, Key Laboratory of Thrombosis and Hemostasis, Ministry of Health, National Clinical Research Center for Hematological Diseases Soochow University Suzhou China

**Keywords:** Iloprost, PKA activity, platelet apoptosis, platelet function, thrombosis and haemostasis

## Abstract

Iloprost, a prostacyclin (PGI_2_) analogue, stimulates the IP receptor (PTGIR) to interact with the Gsα β/γ complex, leading to the activation of adenylate cyclase, which enzyme produces the second messenger cAMP. Elevation in cAMP triggers intracellular signalling events and regulates a wide variety of cellular activities. Thus, we evaluated the effects of Iloprost on platelet function and apoptosis and in vivo haemostasis and thrombosis, as well as the underlying mechanisms. Firstly, we showed that Iloprost concentration‐dependently inhibited agonist‐induced P‐selectin exposure, integrin αIIbβ3 activation, platelet aggregation, ATP release, platelet spreading, and clot retraction. Moreover, Iloprost dose‐dependently inhibited FeCl_3_‐induced mouse mesenteric arteriole thrombosis and markedly prolonged the tail bleeding time. Iloprost also concentration‐dependently inhibited mitochondrial membrane potential (ΔΨm) depolarisation and phosphatidylserine (PS) externalisation in platelets, thereby inhibiting platelet apoptosis, and Iloprost at concentrations lower than 2 nM inhibited only platelet apoptosis but not platelet function. Importantly, Iloprost at low doses markedly elevated peripheral platelet counts in GPIbα antibody‐induced immune thrombocytopenia (ITP). Mechanistic studies showed that Iloprost concentration‐dependently antagonised agonist‐induced decline of protein kinase A (PKA) activity and elevation of cytoplasmic Ca^2+^ in platelets, thereby attenuating platelet activation and aggregation. Elevation in PKA activity inhibited dephosphorylation of proapoptotic protein BAD and reduced caspase‐3 activity, thus retarding platelet apoptosis. These data demonstrate that Iloprost dose‐dependently inhibits platelet function and apoptosis by elevating PKA activity. Moderate‐dose Iloprost impairs haemostasis and thrombosis via suppression of platelet function, and low‐dose Iloprost elevates peripheral platelet levels by inhibiting platelet apoptosis while having no effects on platelet function.

## 1 Introduction

Platelet count in peripheral blood is huge, second only to red blood cells, and platelets are easily activated [1]. Platelets play key roles in thrombosis and haemostasis [2]. At sites of vascular injury, free‐flowing platelets are recruited via the interaction between glycoprotein (GP) Ib/V/IX and immobilised von Willebrand factor (vWF). Platelets firmly adhere to the exposed collagen in the sub‐endothelial matrix through the engagement of collagen receptors α2β1 and GPVI, thereby triggering potent inside‐out signals inducing platelet activation and release of agonists, such as ADP and thromboxane A2 (TXA2), as well as activating platelet major integrin αIIbβ3 [3]. Released agonists interact with G protein‐coupled receptors (GPCRs), further leading to integrin inside‐out activation [4, 5, 6]. The interaction of these activated integrins with fibrinogen/vWF can crosslink platelets to make aggregation and thrombus formation. Ligand binding to integrin triggers outside‐in signals, initiating stable adhesion, spreading, clot retraction, and granule secretion, including P‐selectin, which can result in an irreversible platelet plug [7, 8, 9]. However, if this same process occurs in a diseased, sclerotic, or occluded vessel, the resulting platelet thrombus may break away and block the coronary artery, causing a heart attack, or restrict blood supply to the brain, causing a stroke [10, 11]. The mechanisms of thrombus formation have been well‐established knowledge and have contributed to the development of antithrombotic drugs [12, 13].

Iloprost, a PGI_**2**_ analogue, is clinically used to treat primary pulmonary arterial hypertension (PPH) [14, 15], which is a disease or pathophysiological syndrome caused by an abnormal increase in pressure in the pulmonary arteries from known or unknown causes, including a lack of substances that dilate blood vessels and thrombosis in the pulmonary vessels [16, 17]. Iloprost induces elevation of cAMP levels in smooth muscle cells, thereby relaxing smooth muscle and dilating blood vessels. Iloprost stimulates the IP receptor, a member of the GPCRs family, to interact with the Gsα β/γ complex [18], leading to the activation of AC, which enzyme produces the second messenger cAMP [19]. Elevation in cAMP triggers intracellular signalling events and regulates a wide variety of cellular activities [20, 21]. However, the effects of Iloprost on certain platelet functions, especially on thrombosis and haemostasis, have not yet been confirmed.

Platelet apoptosis is an active and programmed cell death, which is also regulated by genes, enzymes, and related signal transduction pathways [22, 23]. Now, accumulating evidence indicates that platelet apoptosis provoked by various pathological stimuli results in shortened platelet life span and thrombocytopenia in many common diseases, such as infections [24, 25], immune thrombocytopenia (ITP) [26], stored platelets, and diabetes [27], as well as during some pharmacological treatments [28]. Moreover, apoptotic and activated platelets are rapidly cleared in vivo. Platelet clearance has different mechanisms, especially with the GPIbα receptor as the centre, through its desialylation, receptor clustering, and ectodomain shedding. Proinflammatory platelets also undergo clearance in different ways. Platelets undergo apoptosis or activation in these pathways [29]. Our prior studies [30] have shown that PKA activity is markedly reduced in platelets during these different pathological processes, and PKA inhibition provokes mitochondria‐mediated intrinsic programmed platelet apoptosis in vitro and rapid platelet clearance in vivo [31, 32]. Importantly, we also demonstrated that PKA activation protected platelets from apoptosis induced by storage or pathological stimuli. PKA activity is regulated by intracellular cAMP levels, which are determined by the balance between synthesis and degradation by adenylate cyclase (AC) [15] and phosphodiesterases [33], respectively. Iloprost triggers AC‐mediated elevation of intracellular cAMP, but its effect on platelet apoptosis has not been carefully studied.

In this study, we carefully evaluated the direct effects of different concentrations of Iloprost on platelet function, platelet apoptosis, and in vivo haemostasis and thrombosis, as well as the underlying mechanisms. We showed that Iloprost dose‐dependently inhibits platelet function and apoptosis by elevating PKA activity. Iloprost at low doses inhibits platelet apoptosis while having no effect on platelet function, suggesting that low‐dose Iloprost may be used for extending platelet life span or elevating peripheral platelet levels in stored platelets or in disease conditions relating to exacerbated platelet apoptosis (e.g., immune thrombocytopenia). In addition, Iloprost at moderate doses may be used as an antiplatelet drug for preventing the formation of unwanted thrombi.

## 2 Materials and Methods

### 2.1 Healthy Volunteers

Approval for obtaining whole‐blood samples from 26 healthy volunteers was obtained from the Ethics Committee of the First Affiliated Hospital of Soochow University, and informed consent was obtained from all subjects involved in this study according to the Declaration of Helsinki. All samples were used for scientific research only.

### 2.2 Mice

144 C57BL/6J WT mice were purchased from JOINN Laboratories. Mice used in this study were 6–8 weeks old, and experiments included balanced groups of male and female mice unless otherwise stated. All animal experiments were approved by the Ethics Committee of the First Affiliated Hospital of Soochow University.

### 2.3 Reagents and Antibodies

ADP (#384), Collagen (#385), Thrombin (#386), ATP Standard (#387), and Luciferin/luciferase reagents (#395) were purchased from Chrono‐log Corporation (Havertown, PA, USA). U46619 (538944) was purchased from Calbiochem (La Jolla, CA, USA). Iloprost (HY‐A0096) and Ionomycin (HY‐13434) were purchased from MedChem Express (Princeton, NJ, USA). Calcein‐AM was purchased from Dojindo Laboratories. cAMP Biotrak Enzymeimmunoassay (EIA) System kit (RPN225PL) was purchased from Cytiva (Danaher, USA). PAGE Gel Fast Preparation Kit (PG 113) was purchased from Yazin Biotechnology (Shanghai, China). A mixture of purified rat monoclonal antibodies against mouse GPIbα (R300) was purchased from Emfret Analytics (Germany). 10 × Lysis Buffer (9803) and antibodies for western blotting against GAPDH (2118), β‐actin (4970), caspase‐3 (9662), VASP (3112), and phospho‐VASP (Ser157) (3111) were purchased from Cell Signalling Technology (Beverly, MA, USA). FITC‐conjugated anti‐P‐selectin antibody (304904) was purchased from BioLegend (San Diego, CA, USA). Phospho‐BAD (Ser155) (ab28825) was purchased from Abcam (Cambridge, MA, UK). FITC‐conjugated PAC‐1 (340507) was purchased from BD Biosciences (San Jose, CA, USA). Rat IgG (A7031), N‐[2‐((p‐Bromo Cinnamyl) amino)ethyl]‐5‐isoquinoline sulfonamide (H89) (S1644), JC‐1 (C2005) and Fluo‐4 AM (S1060) were purchased from Beyotime Institute of Biotechnology (Beyotime, Shanghai, China). FITC‐conjugated Lactadherin (BLAC‐FITC) was purchased from Haematologic Technologies (Essex Junction, VT, USA).

### 2.4 Platelet Counts and Preparation

Platelet and blood cell counts were performed with the Sysmex XP‐100 Haematologic Analyser (Sysmex Corporation). Washed platelets from healthy volunteers were prepared as previously described [30]. Briefly, whole blood was drawn from the cubital vein and anticoagulated with a 1:7 volume of acid–citrate–dextrose (ACD: 2.5% trisodium citrate, 2.0% D‐glucose, 1.5% citric acid). Platelet‐rich plasma (PRP) was collected from the whole blood by centrifugation at 200 × g for 11 min. Platelets were washed twice with CGS buffer (0.123 M NaCl, 0.033 M D‐glucose, 0.013 M trisodium citrate, pH 6.5), resuspended in modified Tyrode's buffer (MTB) (2.5 mM Hepes, 150 mM NaCl, 2.5 mM KCl, 12 mM NaHCO_3_, 5.5 mM D‐glucose, 1 mM CaCl_2_, 1 mM MgCl_2_, pH 7.4) to a final concentration of 3 × 10^8^/mL, and allowed to incubate at 22°C for 1–2 h. For the preparation of murine platelets, whole blood from mice was collected from the coeliac vein using a 1:7 volume of ACD as an anticoagulant. Platelets were washed with CGS buffer, resuspended in MTB to a concentration of 1 × 10^9^/mL, and allowed to incubate at 22°C for 1–2 h. For preparation of PRP, whole blood was anti‐coagulated with a 1:9 volume of 3.8% trisodium citrate.

### 2.5 Flow Cytometry Analysis

After the pretreated platelets were incubated with FITC‐labelled P‐selectin antibody (10 μg/mL) or FITC‐labelled PAC‐1 (25 μg/mL) for 15 min, JC‐1 (2 μg/mL) for 5 min, FITC‐labelled lactadherin (10 μg/mL) for 20 min, or Fluo‐4/AM (2 mM) for 10 min at room temperature in the dark, the reaction was stopped by adding MTB, and the samples were analysed by a flow cytometer (FC 500, Beckman‐Coulter).

### 2.6 Platelet Aggregation and Secretion

Platelet aggregation and secretion were recorded in a Chrono‐Log lumi‐aggregometer under stirring conditions (1200 rpm) at 37°C. The pretreated platelets (3 × 10^8^/mL) were stimulated with different agonists. Luciferin/luciferase (10 μL) was added to 240 μL of pretreated platelet suspension within 1 min before stimulation. Platelet aggregation and secretion were monitored continuously over 5–10 min.

### 2.7 Platelet Spreading on Immobilised Fibrinogen

Adhesive slides were coated with 50 μg/mL fibrinogen in 0.1 M NaHCO_3_ (pH 8.3) at 4°C overnight. Washed platelets (2 × 10^7^/mL) were allowed to adhere to and spread on fibrinogen‐coated wells at 37°C for 1 or 2 h with stimulation by thrombin. After washing, the cells were fixed, permeabilized, and blocked with 5% BSA and stained with Alexa Fluor 488‐conjugated phalloidin. Adherent platelets were viewed with an Olympus FluoView FV1000 confocal microscope. Images were acquired, and the spreading area of single platelets was measured using ImageJ2x software, with pixel number as the unit of size. Five randomly selected fields from at least three different tests were used for statistical analysis.

### 2.8 Clot Retraction

Washed platelets (4 × 10^8^/mL) were resuspended in modified Tyrode's buffer in clean aggregometer tubes and mixed with 150 μg/mL purified human fibrinogen. The clots were initiated by the addition of thrombin to a final concentration of 0.1 U/mL, followed by incubation at 37°C for 60 min. Clot retraction was monitored every 5 min and photographed. Clot size was quantified from photographs using Image J2x software.

### 2.9 In Vivo Thrombosis Model

In the ferric chloride (FeCl_3_)‐induced mesenteric arteriole thrombosis model experiment, platelets were isolated from donor mice and labelled with calcein‐AM (5 μg/mL). After oral administration of different doses of Iloprost for 20 min in male mice, the recipient mice were anaesthetised with 2% pentobarbital (100 mg/kg, i.p.). Male mice were injected i.v. with calcein‐labelled platelets (5 × 10^6^ /g) of matching genotype. The mesentery vascular bed was exteriorised, and one arteriole was chosen and visualised and recorded with an inverted fluorescent microscope (Leica Microsystems). Thrombus formation was induced by topical application of a 1‐mm^2^ filter paper soaked with 6% FeCl_3_. The vessel occlusion time was defined as the time to complete cessation of blood flow.

### 2.10 Haematologic Analysis and Tail Bleeding Time

Whole blood cell counts were performed with a Sysmex XP‐100 Haematologic Analyser. In the tail bleeding time experiments, 6–8 weeks old mice (equivalent numbers of males and females) were anaesthetised with 2% pentobarbital (100 mg/kg, i.p.). Tails were amputated 4 mm from the tip and immediately immersed into saline kept at 37°C. For each tail, the bleeding time from the tail transection to the moment the blood flow stopped for more than 1 min (i.e., no rebleeding within 60 s), or a maximum of 600 s, was recorded.

### 2.11 cAMP Assay

The pretreated platelets (3 × 10^8^/mL) were centrifuged, resuspended in lysis reagent for 10 min and subjected to cAMP assay using the cAMP enzyme immunoassay (EIA) system kit (Cytiva) according to the manufacturer's instructions.

### 2.12 Western blotting

The pretreated platelets (3 × 10^8^/mL) were lysed with 10 × lysis buffer containing protease inhibitor and phosphatase inhibitor on ice for 15 min. Proteins were separated by SDS‐PAGE, transferred onto PVDF membranes, and then subjected to immunoblotting. Protein bands were visualised by the ECL Chemiluminescence System on Kodak film. Quantification was performed with ImageJ software.

### 2.13 Statistical Analysis

All of the reported figures and data are derived from at least three independent experiments. All data are expressed as mean ± SD. Numeric data were analysed using one‐way or two‐way ANOVA followed by Dunnett's multiple comparisons test. The significance of data was assessed using GraphPad Prism 9 software. For all analyses, a *p* < 0.05 was considered to indicate statistical significance.

## 3 Results

### 3.1 Iloprost Concentration‐Dependently Attenuates Platelet Activation

**FIGURE 1:**
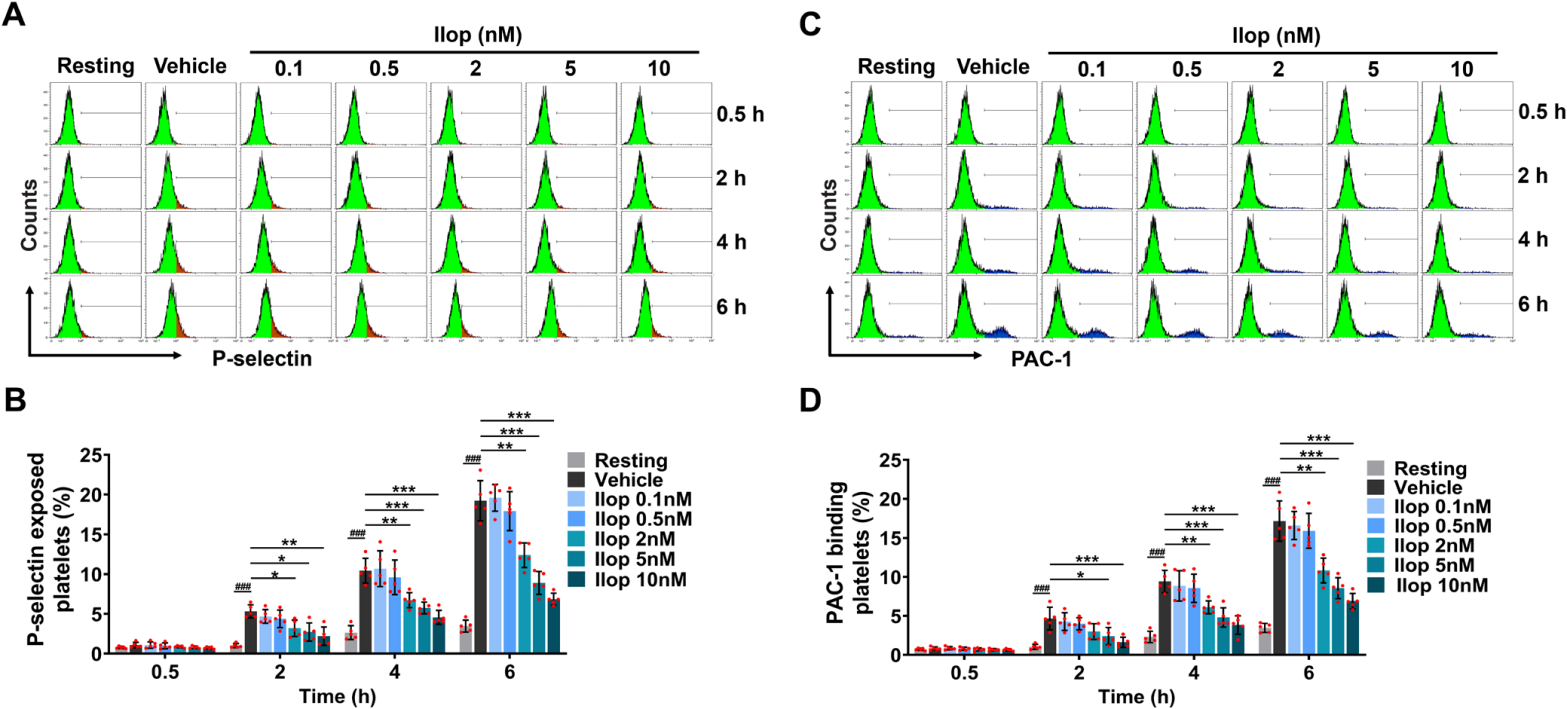
Iloprost (Ilop) concentration‐dependently attenuates platelet activation. (A–D) Washed platelets from healthy human volunteers were pretreated with different concentrations of Iloprost (Ilop) (0.1, 0.5, 2, 5 and 10 nM) or 0.1% DMSO (vehicle control and resting platelets) at 37°C for 5 min, followed by activation with 10 μg/mL SZ‐2 or mouse IgG (resting platelets) at 37°C. The time course of platelet activation occurring in human platelets was analysed by flow cytometry. (A, B) Iloprost inhibited P‐selectin exposure (CD62P) during α‐granule secretion. Representative flow cytometric figures (A) and quantification (B) of platelet P‐selectin expression on platelet surface. (C, D) Iloprost inhibited integrin αIIbβ3 activation. Representative flow cytometric figures (C) and quantification (D) of platelet αIIbβ3 activation (PAC‐1 binding) on platelet surface. Data are expressed as mean ± SD (*n* = 5). ^#^ *p* < 0.05, ^##^ *p* < 0.01, ^###^ *p* < 0.001 compared to resting platelets, *p < 0.05, **p < 0.01, ***p < 0.001 compared to vehicle control, by two‐way ANOVA followed by Dunnett's multiple comparisons test.

We first evaluated the effects of Iloprost on platelet activation. P‐selectin is a glycoprotein that exists on the α‐granule membrane of stationary platelets and is expressed on the surface of platelets after stimulation by activation signals, which is a characteristic indicator of platelet activation. We pretreated healthy human platelets with different concentrations (0.1, 0.5, 2, 5 and 10 nM) of Iloprost and subsequently stimulated the platelets with a well‐characterised antiplatelet glycoprotein (GP) Ibα monoclonal antibody SZ‐2 [31, 32, 34, 35, 36] before P‐selectin exposure on platelets was detected by flow cytometry. We found that P‐selectin exposure on platelets stimulated by SZ‐2 binding in vehicle control significantly increased compared to that in resting platelets with prolonged incubation time of SZ‐2. Iloprost at a concentration of 2 nM or higher markedly reduced P‐selectin exposure on SZ‐2‐stimulated platelets compared with vehicle control, and as the concentrations and incubation times of Iloprost increased, Iloprost presented a stronger inhibitory effect on P‐selectin exposure on platelets (Figure 1A,B), so did other prostacyclin analogues, such as Beraprost and Treprostinil (Figure S1A,B). And, in all examined groups, the inhibitory effect of Iloprost on P‐selectin exposure followed a concentration‐dependent manner, with the strongest inhibitory effect seen at 10 nM. These data indicate that Iloprost concentration‐dependently inhibited P‐selectin exposure during α‐granule secretion on platelets.

Different platelet activation signalling pathways will converge into common signalling events that ultimately induce the inside‐out signalling process dependent on conformational changes of activated integrin αIIbβ3 [37, 38]. Thus, we detected the activation of αIIbβ3 and found that, consistent with the inhibitory effects of Iloprost on P‐selectin exposure, Iloprost concentration‐dependently suppressed αIIbβ3 activation on platelets, as indicated by PAC‐1 (an activation‐induced conformational epitope on αIIbβ3) binding on platelets (Figure 1C,D), so were other prostacyclin analogues, such as Beraprost and Treprostinil (Figure S1C,D). Taken together, these data indicate that Iloprost attenuates platelet activation in a concentration‐dependent manner.

### 3.2 Iloprost Attenuates Platelet Aggregation and Dense Granule Secretion

**FIGURE 2:**
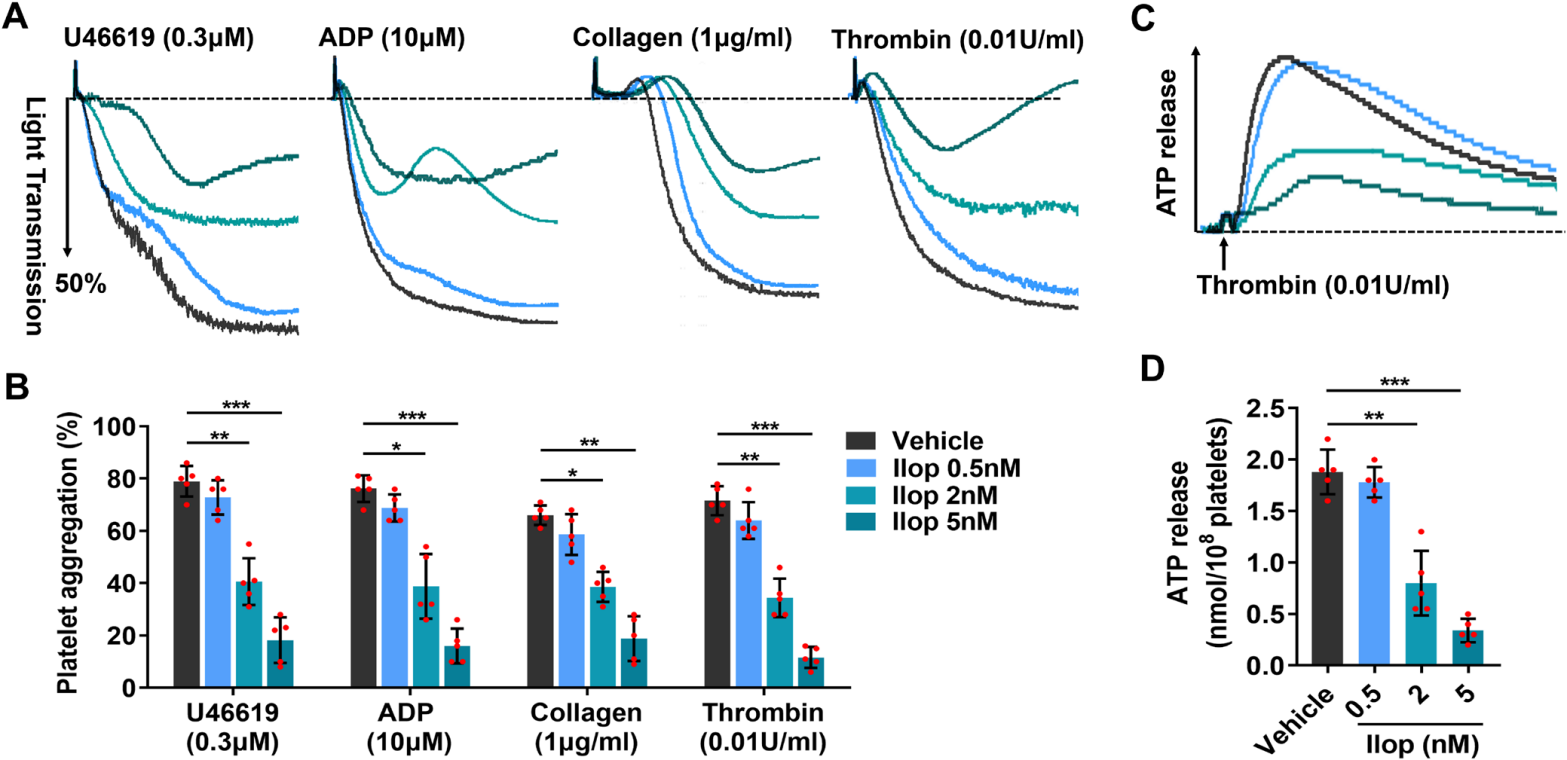
Iloprost attenuates platelet aggregation and dense granule secretion. (A–D) Washed platelets (WP) or platelet‐rich plasma (PRP) from healthy human volunteers were pretreated with different concentrations of Iloprost (0.5, 2 and 5 nM) or 0.1% DMSO (vehicle control) at 37°C for 20 min, followed by activation with different agonists under constant stirring. (A, B) Iloprost concentration‐dependently attenuated platelet aggregation induced by U46619 (0.3 μM in WP), ADP (10 μM in PRP), collagen (1 μg/mL in WP), or thrombin (0.01 U/mL in WP). Representative aggregation tracings (A) and summary data (B) from five experiments are presented. (C, D) Iloprost concentration‐dependently decreased ATP release stimulated by thrombin (0.01 U/mL in WP). Representative results (C) and summary data (D) of five experiments are presented. Data are expressed as mean ± SD (*n* = 5). *p < 0.05, **p < 0.01, ***p < 0.001 compared to vehicle control, by one‐way ANOVA followed by Dunnett's multiple comparisons test.

Platelet aggregation and release play pivotal roles in haemostasis and thrombosis. To investigate the effects of Iloprost on platelet aggregation and release, platelet‐rich plasma (PRP) or washed platelets (WP) from healthy human volunteers were preincubated with different concentrations (0.5, 2 and 5 nM) of Iloprost for 20 min, followed by activation with different agonists. Platelet aggregation and ATP release during dense granule secretion were measured simultaneously using a luciferin‐luciferase luminescence assay. We found that 2 or 5 nM Iloprost presented the inhibitory effect on platelet aggregation and ATP release platelets in different agonist‐stimulated platelets compared with vehicle control, and as the concentrations of Iloprost increased, Iloprost concentration‐dependently attenuated platelet aggregation induced by U46619, ADP, collagen, or thrombin (Figure 2A,B) and decreased ATP release stimulated by thrombin (Figure 2C,D). And, in all examined groups, 5 nM Iloprost exhibited the strongest inhibitory effect on platelet aggregation and ATP release. These data demonstrate that Iloprost attenuates platelet aggregation and dense granule secretion in a concentration‐dependent manner.

### 3.3 Iloprost Impairs Platelet Spreading and Clot Retraction

**FIGURE 3:**
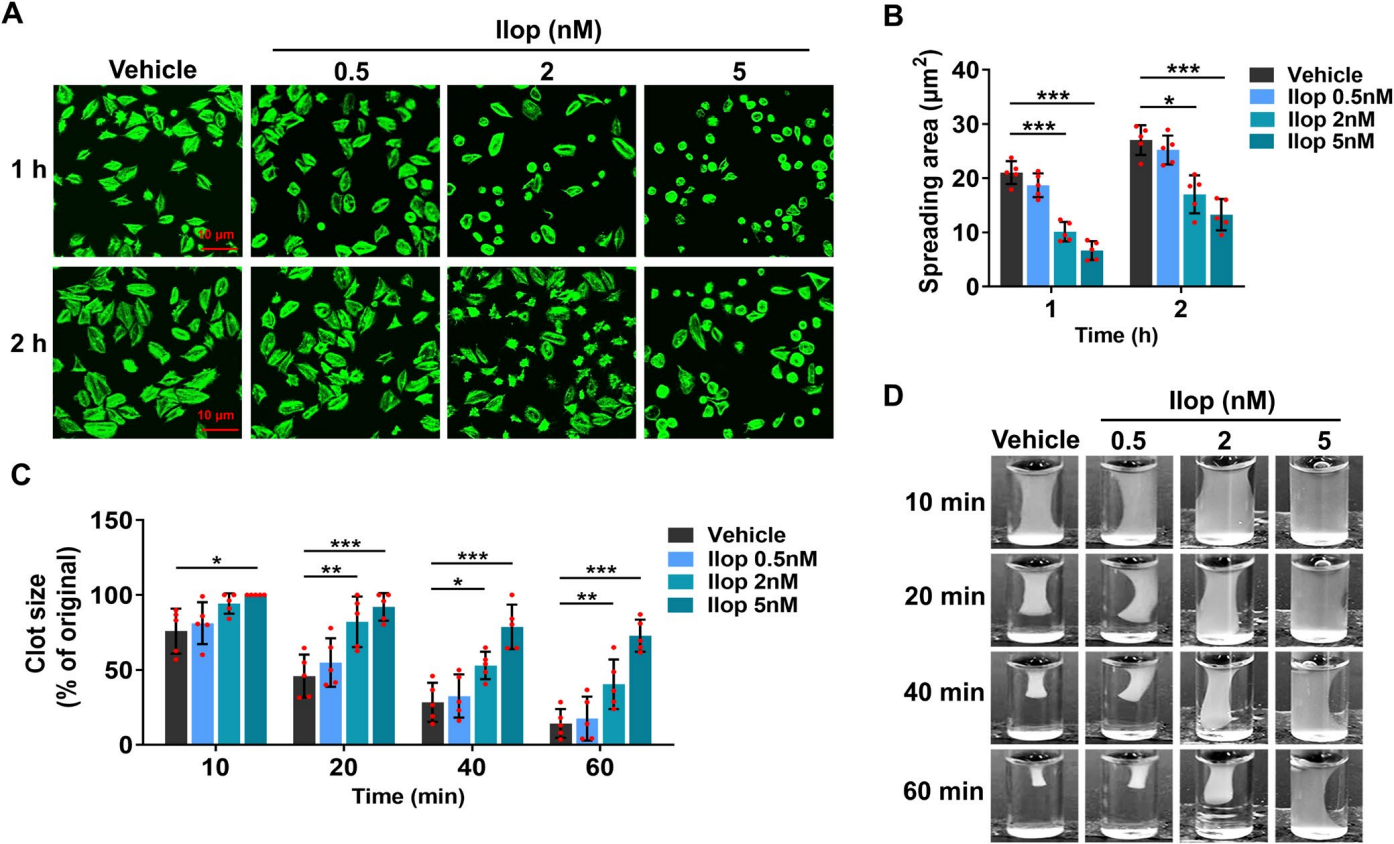
Iloprost impairs platelet spreading and clot retraction. (A, B) Iloprost inhibited platelet spreading on immobilised fibrinogen. Washed platelets from healthy human volunteers were preincubated with different concentrations of Iloprost (0.5, 2 and 5 nM) or 0.1% DMSO (vehicle control) for 5 min, and then allowed to adhere and spread on fibrinogen‐coated wells by thrombin (0.01 U/mL) stimulation at 37°C for 1 or 2 h. After being fixed, permeabilized, and stained, the platelets were observed with a fluorescence microscope. Images were acquired, and the spreading area of single platelets was measured using ImageJ2x software, with pixel number as the unit of size. (A) Representative images and (B) Surface areas of single platelets from 5 randomly selected fields of five different tests. (C, D) Iloprost inhibited clot retraction induced by thrombin. Washed human platelets in modified Tyrode's buffer were preincubated with different concentrations of Iloprost (0.5, 2 and 5 nM) or 0.1% DMSO (vehicle control) for 5 min, and then mixed with purified human fibrinogen (150 μg/mL). The clots were initiated by the addition of thrombin (0.1 U/mL) and incubation at 37°C. (C) Representative images of clot retraction at all observed time points from five separate experiments. (D) 2D retraction of clots was measured, and the data are expressed as the clot size (% of the initial clot). Data are expressed as mean ± SD (*n* = 5). *p < 0.05, **p < 0.01, ***p < 0.001 compared to vehicle control, by two‐way ANOVA followed by Dunnett's multiple comparisons test.

Activated integrin αIIbβ3 binds to fibrinogen and transduces signals into platelets (outside‐in signalling), triggering a cascade of signal reactions, which initiate spreading and clot retraction to stabilise arterial thrombus formation [37, 39]. To test the effects of Iloprost on integrin outside‐in signalling‐dependent platelet spreading and clot retraction, washed platelets from healthy human volunteers were preincubated with different concentrations (0.5, 2 and 5 nM) of Iloprost for 5 min, followed by stimulation with thrombin. We found that, in line with the inhibitory effects of Iloprost on αIIbβ3 activation, 2 or 5 nM Iloprost markedly suppressed platelet spreading on fibrinogen and attenuated clot retraction in the presence of thrombin compared with vehicle control, and as the concentrations of Iloprost increased, Iloprost concentration‐dependently reduced platelet spreading on fibrinogen (Figure 3A,B) and clot retraction (Figure 3C,D) at all observed time points. And, at all examined groups, 5 nM Iloprost exhibited the strongest inhibitory effect on platelet spreading on fibrinogen and clot retraction. These data demonstrate that Iloprost impairs platelet spreading and clot retraction in a concentration‐dependent manner.

### 3.4 Iloprost Dose‐Dependently Attenuates Thrombosis and Haemostasis by Inhibiting Platelet Function

**FIGURE 4:**
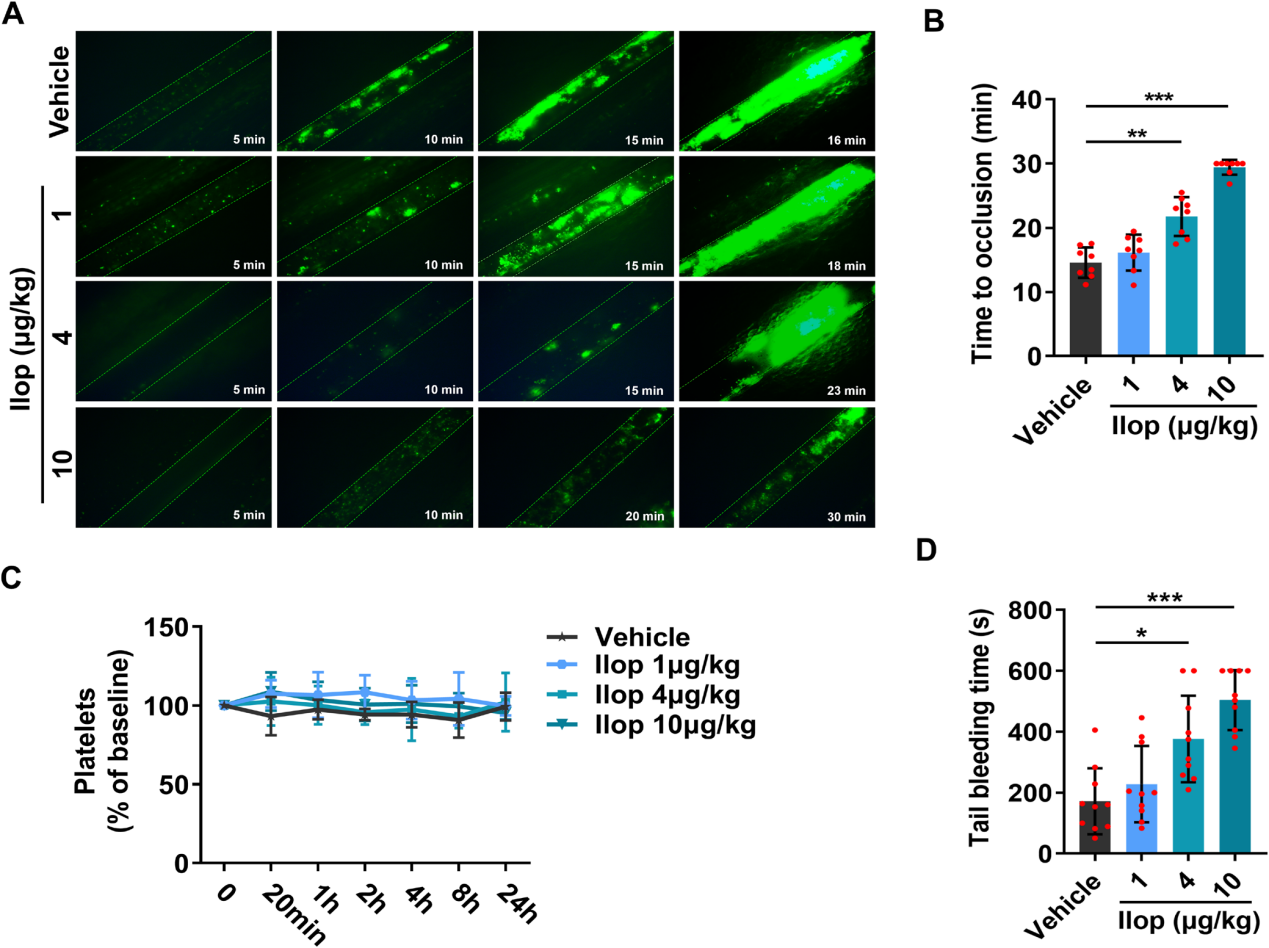
Iloprost dose‐dependently attenuates haemostasis and thrombosis by inhibiting platelet function. (A, B) Iloprost inhibited thrombus formation in mesenteric arterioles of wild‐type mice. After oral administration of different doses of Iloprost (0, 1, 4 and 10 μg/kg) for 20 min in wild‐type mice, FeCl_3_‐induced thrombus formation was performed and recorded by real‐time microscopy. (A) Representative images of FeCl_3_‐induced mesenteric arteriole thrombosis in wild‐type mice. Time after FeCl_3_‐induced injury is indicated at the bottom right of each image. (B) Occlusion time of the mesenteric arteriole as measured by FeCl_3_ injury in wild‐type mice. (C) Iloprost has no changes in peripheral platelet counts. Platelet counts were examined at the indicated time points after oral administration of different doses of Iloprost (0, 1, 4 and 10 μg/kg) in wild‐type mice. (D) Iloprost dose‐dependently attenuates haemostasis, as indicated by tail bleeding time. After oral administration of different doses of Iloprost (0, 1, 4 and 10 μg/kg) for 20 min in wild‐type mice, tail‐ bleeding experiment was performed and recorded. Data are expressed as mean ± SD. *p < 0.05, **p < 0.01, ***p < 0.001 compared to vehicle control, by one‐ or two‐way ANOVA followed by Dunnett's multiple comparisons test.

Having demonstrated the inhibitory effect of Iloprost on platelet function in vitro, we sought to determine the potential implications of Iloprost on thrombosis and haemostasis. We first investigated the effect of Iloprost on arterial thrombosis in vivo using a FeCl_3_‐injured mouse mesenteric arteriole thrombosis model. After oral administration of different doses of Iloprost (1, 4 and 10 μg/kg) in mice for 20 min, FeCl_3_‐induced thrombus formation was performed and observed at different time points by fluorescence microscopy. We found that as the doses of oral administration of Iloprost increased, mice exhibited delayed and diminished thrombus formation, and mean occlusion time became significantly longer in the mice compared with vehicle control, and in all observed groups, 10 μg/kg Iloprost exhibited the strongest antithrombotic effect (Figure 4A,B). These data demonstrate that Iloprost attenuates thrombosis in a dose‐dependent manner.

Antithrombotic medications often come with an inherent trade‐off—increased susceptibility to bleeding complications [40, 41]. To investigate the effect of Iloprost on haemostasis in vivo, we performed a dose‐dependent tail bleeding experiment and examined tail bleeding time of mice at 20 min after oral administration of different doses of Iloprost (1, 4 and 10 μg/kg). We found that as the doses of Iloprost increased, Iloprost markedly prolonged the tail bleeding time compared with vehicle control, and at all observed groups, 10 μg/kg Iloprost presented the longest bleeding time (in 40% of oral administration of 10 μg/kg Iloprost mice, bleeding persisted during a 10‐min observation period.), but all the mice exhibited no tendency toward spontaneous bleeding (Figure 4D). These data indicate that Iloprost attenuates haemostasis in a dose‐dependent manner. Furthermore, we examined the changes of platelet counts after oral administration of different doses of Iloprost (1, 4 and 10 μg/kg) in wild‐type mice. We found that whether in high or low doses, Iloprost has no effects on platelet counts at all examined time points (Figure 4C).

Taken together, these data indicate that Iloprost dose‐dependently attenuates thrombosis and haemostasis by inhibiting platelet function rather than reducing platelet count.

### 3.5 Iloprost Attenuates Platelet Activation and Aggregation by Elevating PKA Activity

**FIGURE 5:**
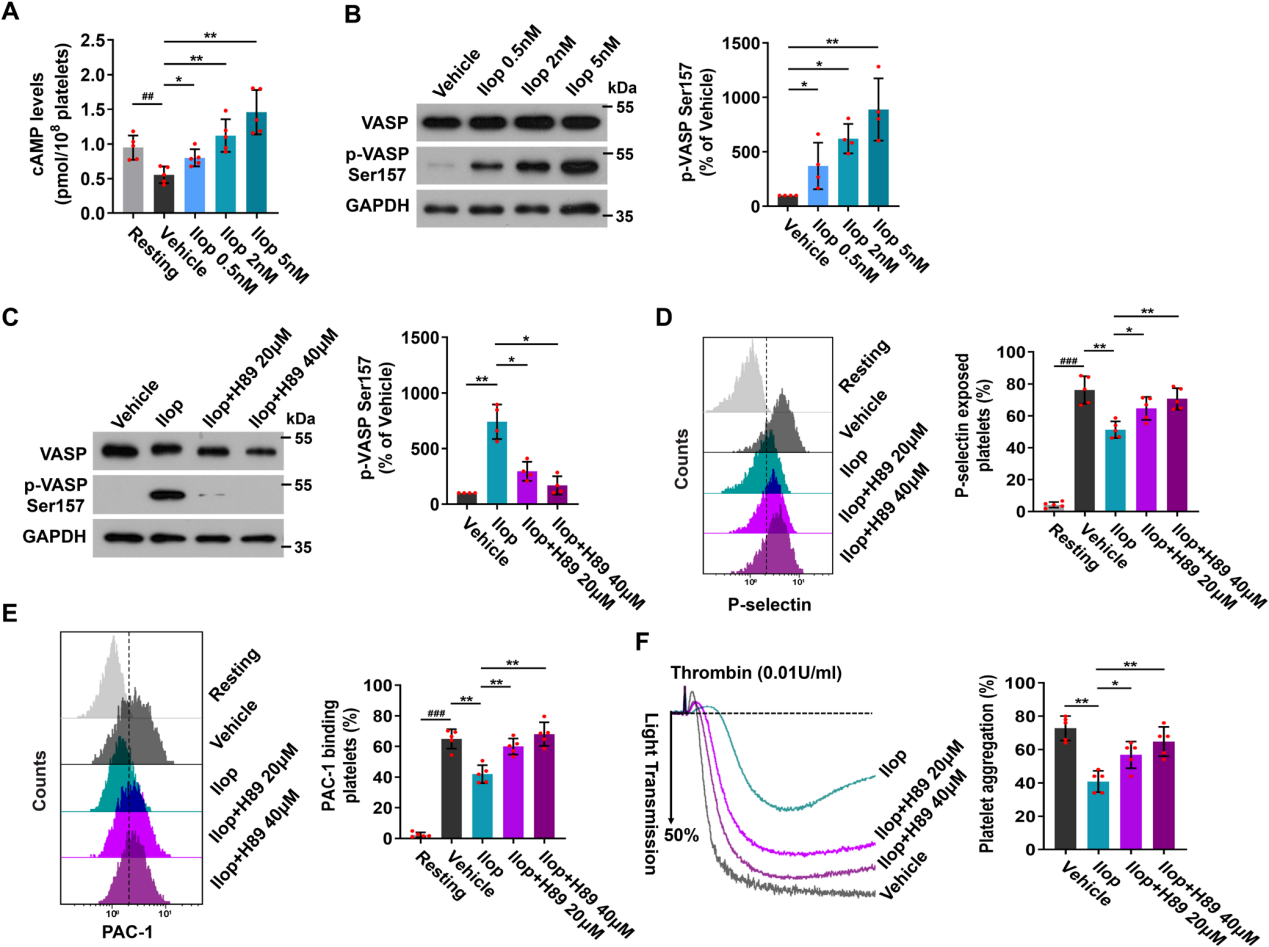
Iloprost attenuates platelet activation and aggregation by elevating PKA activity. (A, B) Washed platelets from healthy human volunteers were pretreated with different concentrations of Iloprost (0.5, 2 and 5 nM) or 0.1% DMSO (vehicle control and resting platelets) at 37°C for 20 min, and then followed by stimulation with thrombin (0.01 U/mL). (A) Iloprost concentration‐dependently antagonised intracellular cAMP decrease in thrombin‐stimulated platelets. cAMP levels from five separate experiments were measured by the enzyme immunoassay (EIA) system. (B) Iloprost concentration‐dependently enhanced phosphorylation of VASP at Ser157. Western blot analysis of VASP phosphorylation with anti‐p‐VASP Ser157 antibody. Representative immunoblots and densitometry of immunoblots for p‐VASP Ser157 from four independent experiments are shown. (C–F) Washed platelets from healthy human volunteers were pretreated with Iloprost (2 nM) or 0.1% DMSO (vehicle control and resting platelets) at 37°C for 20 min and then incubated with H89 (20 or 40 μM) at 37°C for 20 min, followed by stimulation with thrombin (0.01 U/mL). (C) H89 markedly reduced phosphorylation of VASP at Ser157 enhanced by Iloprost. Western blot analysis of VASP phosphorylation with anti‐p‐VASP Ser157 antibody. Representative immunoblots and densitometry of immunoblots for p‐VASP Ser157 from four independent experiments are shown. (D, E) H89 reversed P‐selectin exposure and integrin αIIbβ3 activation attenuated by Iloprost. Flow cytometry analysis of P‐selectin exposure (D) and integrin αIIbβ3 activation (E). Representative flow cytometric figures and quantification of platelet P‐selectin expression and integrin αIIbβ3 activation (PAC‐1 binding) from five experiments are shown. (F) H89‐reversed platelet aggregation attenuated by Iloprost. Representative aggregation tracings and summary data from five experiments are presented. Data are expressed as mean ± SD. ^#^ *p* < 0.05, ^##^ *p* < 0.01, ^###^ *p* < 0.001 compared to resting platelets, *p < 0.05, **p < 0.01, ***p < 0.001 compared to vehicle control, by one‐way ANOVA followed by Dunnett's multiple comparisons test.

We further investigated the mechanism of Iloprost attenuating platelet function. Washed platelets from healthy human volunteers were preincubated with different concentrations (0.5, 2 and 5 nM) of Iloprost for 20 min, followed by activation with thrombin, and then intracellular cAMP levels were measured by EIA. We found that intracellular cAMP decreased dramatically when platelets were activated by thrombin. However, as the concentrations of Iloprost increased, Iloprost concentration‐dependently antagonised intracellular cAMP decrease in thrombin‐stimulated platelets (Figure 5A). PKA is regulated by intracellular cAMP levels and the direct effector of cAMP signalling [15, 42]. We detected PKA activity, which is indicated by phosphorylation of the PKA substrates vasodilator‐associated stimulated phosphoprotein (VASP) at Ser157 [43], and found that in line with the effects of Iloprost on intracellular cAMP, Iloprost concentration‐dependently elevated PKA activity in thrombin‐stimulated platelets (Figure 5B).

Next, using the PKA inhibitor H89 to treat Iloprost‐treated platelets, we detected PKA activity and platelet activation and aggregation induced by thrombin. We found that H89 markedly reduced PKA activity elevated by Iloprost (Figure 5C), and at the same time H89 reversed P‐selectin exposure during α‐granule secretion, integrin αIIbβ3 activation, and platelet aggregation attenuated by Iloprost (Figure 5D–F).

Taken together, these data indicate that Iloprost attenuates platelet activation and aggregation by elevating PKA activity.

### 3.6 Iloprost Inhibits Platelet Activation and Aggregation by Attenuating Cytoplasmic Ca^**2**+^ Levels Increase

**FIGURE 6:**
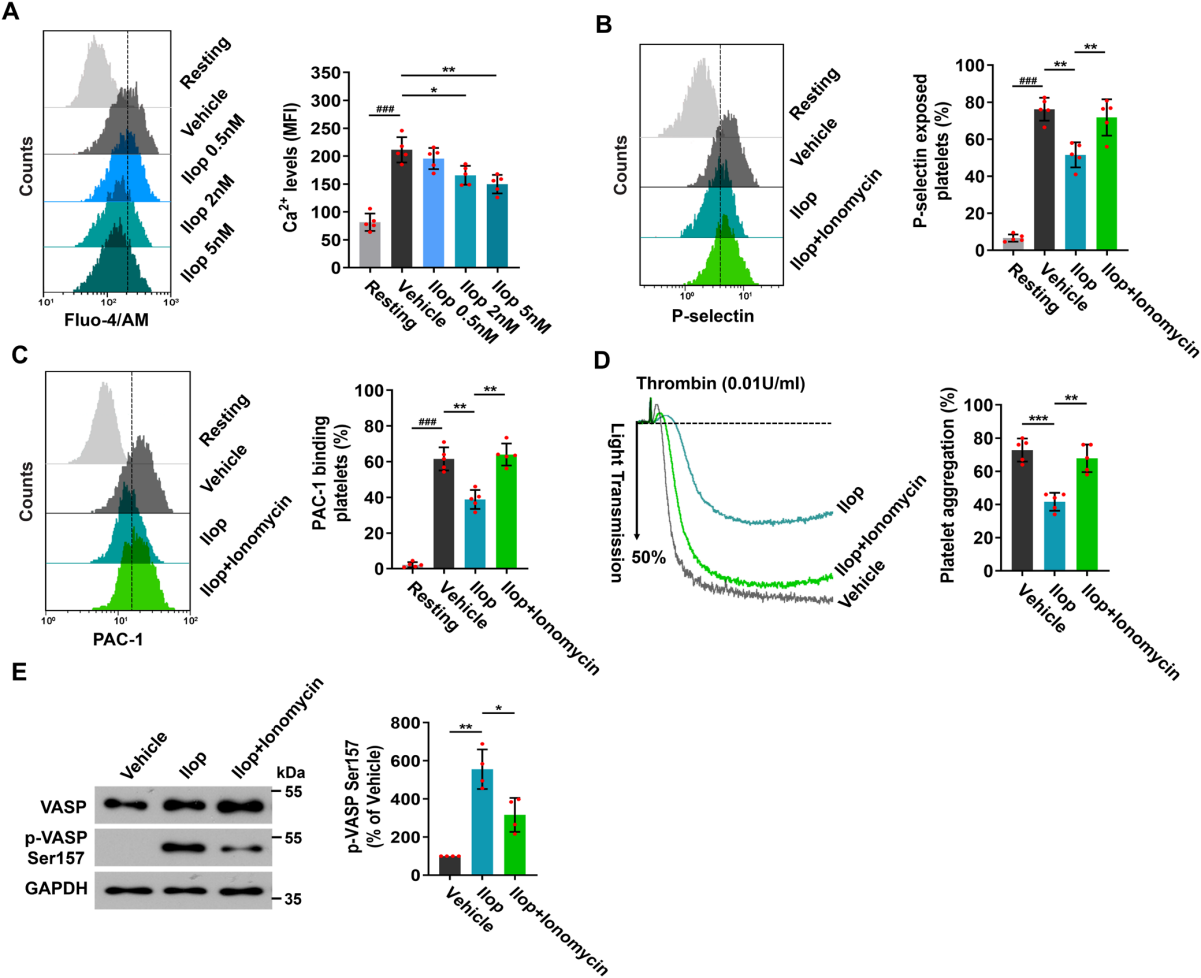
Iloprost inhibits platelet activation and aggregation by attenuating cytoplasmic Ca^2+^ levels increase. (A) Iloprost concentration‐dependently attenuated the increase of cytoplasmic Ca^2+^ levels in thrombin‐stimulated platelets. Washed platelets from healthy human volunteers were pretreated with different concentrations of Iloprost (0.5, 2 and 5 nM) or 0.1% DMSO (vehicle control and resting platelets) at 37°C for 20 min, and then followed by stimulation with thrombin (0.01 U/mL). Ca^2+^ levels from five separate experiments were analysed by flow cytometry with Fluo‐4/AM (a cell‐permeable Ca^2+^ indicator). Representative flow cytometric figures and summary data of Ca^2+^ levels from five experiments are shown. (B–E) Washed platelets from healthy human volunteers were pretreated with Iloprost (2 nM) or 0.1% DMSO (vehicle control and resting platelets) at 37°C for 20 min, and then incubated with Ionomycin (calcium ionophore, 0.3 μM) at 37°C for 10 min, followed by stimulation with thrombin (0.01 U/mL). (B, C) Ionomycin reversed P‐selectin exposure and integrin αIIbβ3 activation was attenuated by Iloprost. Flow cytometry analysis of P‐selectin exposure (B) and integrin αIIbβ3 activation (C). Representative flow cytometric figures and quantification of platelet P‐selectin expression and integrin αIIbβ3 activation from five experiments are shown. (D) Ionomycin reversed platelet aggregation attenuated by Iloprost. Representative aggregation tracings and summary data from five experiments are presented. (E) Ionomycin in part reduced phosphorylation of VASP at Ser157 enhanced by Iloprost. Western blot analysis of VASP phosphorylation with anti‐p‐VASP Ser157 antibody. Representative immunoblots and densitometry of immunoblots for p‐VASP Ser157 from four independent experiments are shown. Data are expressed as mean ± SD. ^#^ *p* < 0.05, ^##^ *p* < 0.01, ^###^ *p* < 0.001 compared to resting platelets, *p < 0.05, **p < 0.01, ***p < 0.001 compared to vehicle control, by one‐way ANOVA followed by Dunnett's multiple comparisons test.

Elevation of cytoplasmic Ca^2+^ levels is critical to numerous steps of platelet function [44]. We investigated the effects of Iloprost on cytoplasmic Ca^2+^ levels. Washed platelets from healthy human volunteers were preincubated with different concentrations (0.5, 2 and 5 nM) of Iloprost for 20 min, followed by activation with thrombin, and then cytoplasmic Ca^2+^ levels were measured by Fluo‐4/AM (a cell‐permeable Ca^2+^ indicator) with flow cytometry. We found that cytoplasmic Ca^2+^ levels increased sharply when platelets were activated by thrombin. However, as the concentrations of Iloprost increased, Iloprost concentration‐dependently attenuated the increase of cytoplasmic Ca^2+^ levels in thrombin‐stimulated platelets (Figure 6A), which was in contrast with the results of cAMP and PKA. Next, using Ionomycin (calcium ionophore) to elevate cytoplasmic Ca^2+^ levels in Iloprost‐treated platelets, we detected platelet activation, and aggregation induced by thrombin. We found that Ionomycin reversed P‐selectin exposure during α‐granule secretion, integrin αIIbβ3 activation and platelet aggregation attenuated by Iloprost (Figure 6B–D). Moreover, we also detected PKA activity and found that Ionomycin in part reduced PKA activity elevated by Iloprost (Figure 6E).

Taken together, these data indicate that Iloprost inhibits platelet activation and aggregation through attenuating cytoplasmic Ca^2+^ levels increase. Additionally, there is the possibility of a feedback regulation between cAMP and Ca^2+^ in platelets.

### 3.7 Iloprost Inhibits Platelet Apoptosis by Elevating PKA Activity and Markedly Elevates Peripheral Platelet Counts in GPIbα Antibody‐Induced ITP

**FIGURE 7:**
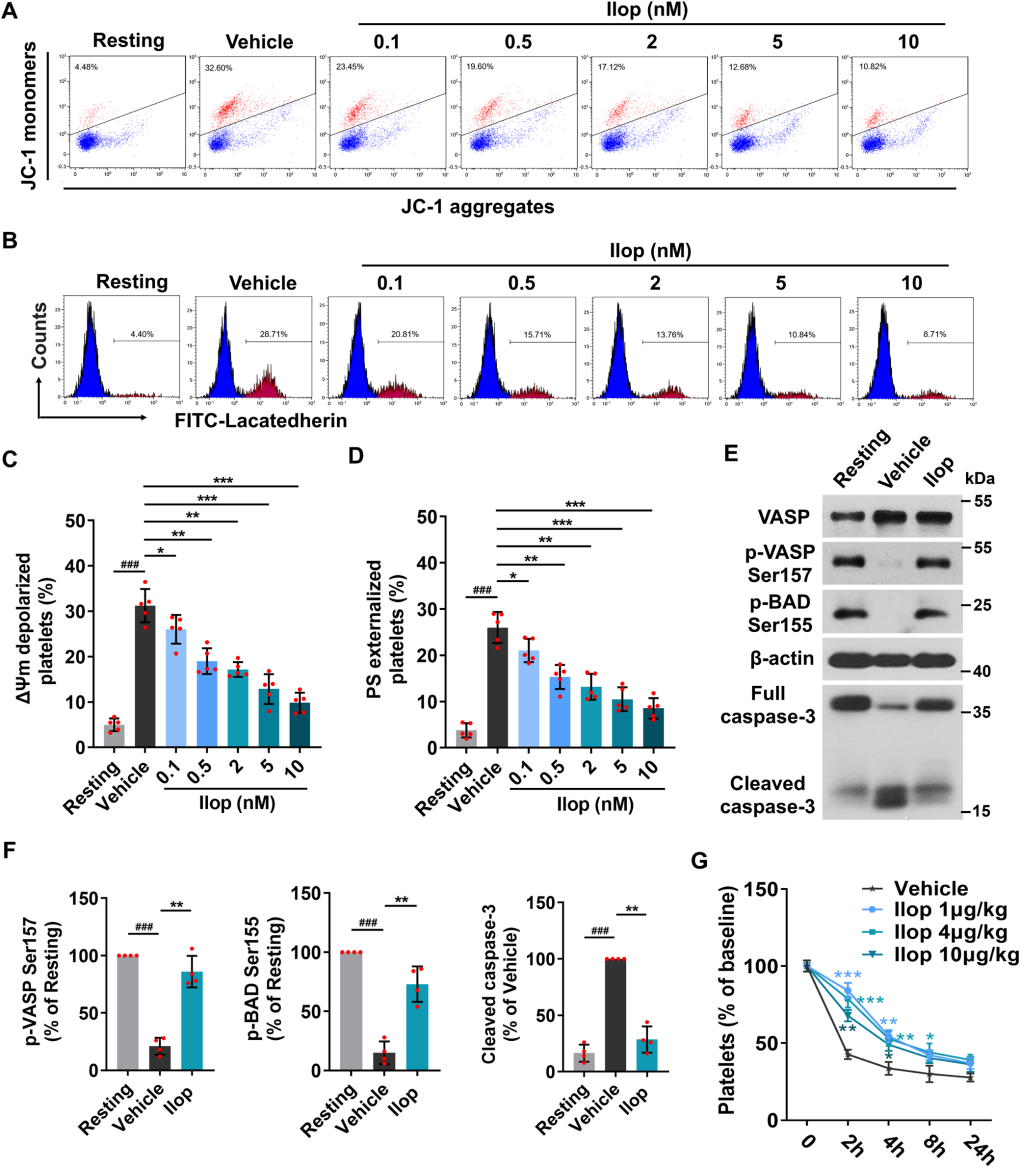
Iloprost inhibits platelet apoptosis by elevating PKA activity. (A–D) Iloprost dose‐dependently inhibited SZ‐2‐induced platelet apoptosis. Washed platelets from healthy human volunteers were pretreated with different concentrations of Iloprost (0.1, 0.5, 2, 5 and 10 nM) or 0.1% DMSO (vehicle control and resting platelets) at 37°C for 5 min, followed by incubation with 10 μg/mL SZ‐2 or mouse IgG (resting platelets) at 37°C for 6 h. Representative flow cytometric figures (A) and quantification (C) of platelet ΔΨm depolarisation. The JC‐1 monomers reflect the monomeric form of JC‐1 that appeared in the cytosol after mitochondrial ΔΨm depolarisation, and the JC‐1 aggregates represent potential‐dependent aggregation in the mitochondria. Representative flow cytometric figures (B) and quantification (D) of platelet PS exposure. PS exposure was detected by FITC‐labelled lactadherin. (E, F) Iloprost elevated PKA activity and enhanced phosphorylation of BAD at Ser155, reducing expression of cleaved caspase‐3. Washed platelets from healthy human volunteers were pretreated with different concentrations of Iloprost (2 nM) or 0.1% DMSO (vehicle control and resting platelets) at 37°C for 5 min, followed by incubation with 10 μg/mL SZ‐2 or mouse IgG (resting platelets) at 37°C for 6 h. Western blot analysis for the levels of indicated proteins with different antibodies. Representative immunoblots (E) and densitometry of immunoblots (F) for indicated proteins from four independent experiments are shown. (G) Iloprost markedly elevates peripheral platelet counts in GPIbα antibody‐induced ITP. Platelet counts were examined at the indicated time points after oral administration of different doses of Iloprost (0, 1, 4 and 10 μg/kg) for 5 min, followed by intraperitoneal injection with 0.1 μg/g R300 (a mixture of purified rat monoclonal antibodies against mouse GPIbα) in wild‐type mice (*n* = 10 per group). Baseline is defined as the platelet counts before oral administration of different doses of Iloprost. Data are expressed as mean ± SD. ^#^ *p* < 0.05, ^##^ *p* < 0.01, ^###^ *p* < 0.001 compared to resting platelets, *p < 0.05, **p < 0.01, ***p < 0.001 compared to vehicle control, by one‐way or two‐way ANOVA followed by Dunnett's multiple comparisons test.

Iloprost activates adenylate cyclase to increase intracellular cAMP levels, which regulates protein kinase A (PKA) activity in platelets. We have previously reported [30] that PKA, as a homeostatic regulator of platelet apoptosis, determines platelet life span and survival. Therefore, we investigated the effect of Iloprost on platelet apoptosis. Washed platelets from healthy human volunteers were pretreated with different concentrations (0.1, 0.5, 2, 5 and 10 nM) of Iloprost, followed by incubation with anti‐GPIbα monoclonal antibodies SZ‐2. We found that consistent with previous reports [31], SZ‐2 induced marked mitochondrial membrane potential (ΔΨm) depolarisation, which initiates mitochondria‐mediated intrinsic programmed apoptosis in platelets [30, 45, 46, 47, 48, 49], and induced phosphatidylserine (PS) externalisation in platelets (Figure 7A–D). Importantly, Iloprost at 0.1 or 0.5 nM, concentrations that were low enough to not affect platelet activation, were able to significantly attenuate SZ‐2 induced ΔΨm depolarisation (Figure 7A,C) and PS exposure (Figure 7B,D). Higher‐concentration Iloprost further attenuated these apoptosis markers. Similar observations were found with other prostacyclin analogues, such as Beraprost and Treprostinil (Figure S2).

To further confirm the mechanism of Iloprost inhibiting platelet apoptosis induced by anti‐GPIbα antibodies SZ‐2, we detected PKA activity and found that PKA activity, as reflected by the Ser157 phosphorylation of the PKA substrates vasodilator‐associated stimulated phosphoprotein (VASP) [43], was obviously reduced in SZ‐2‐treated platelets, which was completely abolished when platelets were pre‐treated with Iloprost (Figure 7E,F). We have previously demonstrated [30, 31] that elevation of PKA activity enhances phosphorylation of proapoptotic protein BAD at Ser155, resulting in inhibition of platelet apoptosis. Consistent with PKA activity results, Iloprost markedly enhanced phosphorylation of BAD at Ser155. Moreover, we detected caspase‐3 activity, which acts as an apoptotic executor and is viewed as the gold standard of platelet apoptosis [50], and found that caspase‐3 activity was significantly elevated in SZ‐2‐treated platelets as indicated by the appearance of 17‐kDa or 19‐kDa fragments, but not in those additionally treated with Iloprost (Figure 7E,F). Together, our results suggest that Iloprost markedly elevates PKA activity, which enhances phosphorylation of BAD at Ser155, reducing caspase‐3 activity, resulting in inhibition of platelet apoptosis.

Our previous studies [31] show that in ITP patients, anti‐GPIbα autoantibodies trigger platelet apoptosis through Akt‐mediated PKA inhibition, leading to markedly reduced peripheral platelet counts. Thus, we examined the changes of peripheral platelet counts after oral administration of different doses of Iloprost (1, 4 and 10 μg/kg) in an anti‐GPIbα antibody‐induced ITP mouse model. Remarkably, Iloprost at 1 μg/kg, a low dose that did not affect arterial thrombosis or bleeding time, was able to significantly relieve anti‐GPIbα antibody‐induced platelet clearance, while moderate (4 μg/kg) or high (10 μg/kg) doses of Iloprost did not exert further enhancement (Figure 7G).

Taken together, these data indicate that Iloprost inhibits platelet apoptosis induced by anti‐GPIbα antibody SZ‐2 through elevation of PKA activity and markedly elevates peripheral platelet counts in GPIbα antibody‐induced ITP.

## 4 Discussion

In this study, we showed that Iloprost directly inhibits agonist‐induced P‐selectin exposure during α‐granule secretion, integrin αIIbβ3 activation, platelet aggregation, dense granule ATP release, platelet spreading, and clot retraction in a concentration‐dependent manner, and that Iloprost dose‐dependently attenuates haemostasis and thrombosis by inhibiting platelet function rather than reducing platelet count. Moreover, Iloprost exhibits its inhibitory effects on platelet function by antagonising intracellular cAMP decrease and thus elevating PKA activity and attenuating cytoplasmic Ca^2+^ levels increase in platelets. In addition, we showed that Iloprost concentration‐dependently inhibits platelet apoptosis by elevating PKA activity, which inhibits dephosphorylation of proapoptotic protein BAD at Ser155, reducing caspase‐3 activity, resulting in inhibition of platelet apoptosis, and markedly elevates peripheral platelet counts in GPIbα antibody‐induced ITP. Collectively, our results indicate that Iloprost concentration‐dependently attenuates platelet function and apoptosis by elevating PKA activity.

Iloprost is clinically used to treat PPH [19, 20], which is caused by known or unknown causes, including a lack of substances that dilate blood vessels and thrombosis in the pulmonary vessels [21]. Therefore, we evaluated the direct effects of Iloprost on thrombosis and haemostasis and on platelet function. GPIbα, the main subunit of the GPIb‐IX complex, contains binding sites for several important ligands, including vWF and thrombin, at the N‐terminal extracellular domain [4, 51]. We and others have previously reported that anti‐GPIbα antibody SZ‐2 activates platelets in vitro [31, 32, 34, 35, 36], which is reproduced in the present study, as shown by the significant P‐selectin exposure during α‐granule secretion and αIIbβ3 activation on platelets after SZ‐2 binding. At all examined groups, 2 nM or higher doses of Iloprost exhibited inhibitory effects on P‐selectin exposure during α‐granule secretion and αIIbβ3 activation on platelets stimulated by SZ‐2 binding in a dose‐dependent manner at all observed time points. We also demonstrated that other prostacyclin analogues, such as Beraprost and Treprostinil, concentration‐dependently inhibited the activation and apoptosis of platelets. However, Iloprost, as the classical stable analogue of prostacyclin, has few side effects and is widely used in clinical practice, especially in stored platelets. Therefore, Iloprost is chosen for further study. Next, we found that in line with the inhibitory effects of Iloprost on platelet activation induced by SZ‐2 binding, platelet aggregation in response to different stimulants of U46619, ADP, collagen, or thrombin; dense granule secretion in response to thrombin; spreading on fibrinogen; and clot retraction were impaired by 2 nM or higher doses of Iloprost in a concentration‐dependent manner. Taken together, Iloprost attenuates platelet function in a concentration‐dependent manner.

As we know, platelets play key roles in thrombosis and haemostasis [1, 2]. Having demonstrated the inhibitory effect of Iloprost on platelet function in vitro, we sought to determine the potential implications of Iloprost on thrombosis and haemostasis. We found that Iloprost dose‐dependently attenuates thrombosis and haemostasis by inhibiting platelet function rather than reducing platelet count. More importantly, all the mice exhibited no tendency toward spontaneous bleeding. Therefore, Iloprost may be used as an antithrombotic medication for preventing the formation of unwanted thrombi.

Iloprost stimulates IP receptors to interact with the Gsα β/γ complex, leading to AC‐mediated elevation of intracellular cAMP [14, 15]. cAMP levels play critical roles in regulating platelet activation [42]. In this study, we found that intracellular cAMP decreased dramatically when platelets were activated by thrombin, and Iloprost concentration‐dependently antagonised intracellular cAMP decrease in thrombin‐stimulated platelets. Elevation of intracellular cAMP attenuated platelet activation. PKA, which is regulated by intracellular cAMP levels and the direct effector of cAMP signalling, phosphorylates a number of proteins to modulate multiple aspects of cellular functions, including platelet activation [14, 42]. Here, we found that Iloprost concentration‐dependently elevated PKA activity, and PKA inhibition reversed platelet activation and aggregation inhibited by Iloprost. Therefore, Iloprost attenuates platelet activation and aggregation by elevating cAMP‐dependent PKA activity.

Elevation of intracellular Ca^2+^ levels is critical to numerous steps of platelet function, including the activation of integrins, shape change, and granule secretion [44]. The elevation of intracellular Ca^2+^ levels is the outcome of intracellular Ca^2+^ release from the DTS (dense tubular system) and influx of extracellular Ca^2+^. Critical early studies demonstrate that cAMP signalling targets both Ca^2+^ entry and intracellular Ca^2+^ release in platelets to modulate numerous aspects of platelet function [52], but the precise mechanisms remain unclear. We further found that Iloprost concentration‐dependently attenuated cytoplasmic Ca^2+^ levels increase in thrombin‐stimulated platelets, and elevation of intracellular Ca^2+^ levels reversed platelet activation and aggregation inhibited by Iloprost, contrary to the results of cAMP and PKA. Moreover, PKA, the direct effector of cAMP signalling, phosphorylates a number of proteins to modulate multiple aspects of platelet function. Thus, cAMP may attenuate the elevation of intracellular Ca^2+^ levels by PKA phosphorylating a number of proteins that inhibit intracellular Ca^2+^ release from the DTS and the influx of extracellular Ca^2+^. Interestingly, we found that elevation of intracellular Ca^2+^ levels, to some degree, attenuated elevation of PKA activity. Hence, there is the possibility of a feedback regulation between cAMP and Ca^2+^.

We have previously reported that PKA, as a homeostatic regulator of platelet apoptosis, determines platelet life span and survival and demonstrated that elevation of PKA activity enhances phosphorylation of proapoptotic protein BAD at Ser155, which reduces the association of BAD with prosurvival protein BCL‐XL on mitochondria [53, 54], thereby enhancing sequestration of proapoptotic proteins BAK and BAX by prosurvival BCL‐XL on mitochondria, resulting in inhibition of platelet apoptosis. Here, Iloprost triggered AC‐mediated elevation of intracellular cAMP, which elevated PKA activity. And PKA activation reduced dephosphorylation of BAD at Ser155, which was consistent with a previous report. Moreover, the gold standard of platelet apoptosis is the evaluation of caspase activity as a functional evidence [50]. Caspase‐3 activity, as an apoptotic executor, was significantly reduced in Iloprost‐treated platelets. Therefore, Iloprost protects platelets from apoptosis by elevating PKA activity, which inhibits dephosphorylation of BAD at Ser155, reducing caspase‐3 activity, resulting in inhibition of platelet apoptosis. Moreover, we found that 0.1 nM or higher Iloprost concentration‐dependently inhibited platelet apoptosis, while 2 nM or higher Iloprost concentration‐dependently attenuated platelet function. Although cAMP‐dependent PKA is a key regulator of both platelet apoptosis and platelet function, different cAMP levels or PKA activity exert different mechanisms. Dose is a key factor for Iloprost to come into play. Here, we showed that Iloprost at doses lower than 2 nM inhibited only platelet apoptosis but not platelet function. Importantly, we found that Iloprost at low doses elevated platelet counts in GPIbα antibody‐induced ITP. Our previous studies [31] show that platelets undergo apoptosis in ITP patients with anti‐GPIbα autoantibodies and demonstrate that anti‐GPIbα antibody binding elicits platelet apoptosis through Akt‐mediated PKA inhibition. Therefore, Iloprost at low doses may be used for elevating peripheral platelet levels by inhibiting platelet apoptosis in GPIbα antibody‐induced ITP while having no effect on platelet function. In addition, we found that low‐dose Iloprost elevated platelet counts more significantly than high‐dose Iloprost in GPIbα antibody‐induced ITP, and that Iloprost at either high or low doses had no effect on platelet counts in healthy mice, although Iloprost inhibited platelet apoptosis. Under normal conditions, platelet production and apoptosis go hand in hand, which keeps the net platelet count in a quasi‐static range. Platelets are blood cells derived from megakaryocytes, but cAMP inhibits megakaryocytic differentiation and platelet release from mature megakaryocytes [55, 56]. Thus, Iloprost may, on the one hand, elevate platelet count by inhibiting platelet apoptosis, while on the other hand suppressing platelet production by elevating cAMP levels, especially Iloprost at high doses. Nevertheless, Iloprost at doses tested in our study may have a minimum effect on megakaryopoiesis. Platelet apoptosis also occurs in the prevention of storage‐dependent platelet damage. Reactive oxygen species (ROS) scavenging or inhibition of ROS production can attenuate platelet apoptosis, which might better preserve the quality of stored platelets, especially for longer storage [57]. In this study, Iloprost at low doses may also be used for extending platelet life span by inhibiting platelet apoptosis in stored platelets. Furthermore, Iloprost is a reversible inhibitor, and its receptor desensitisation is also a reversible phenomenon in human platelets. Long‐term exposure of platelets to high doses of Iloprost might result in desensitisation and internalisation of platelet prostaglandin receptors accompanied by a loss in receptor density on the platelet surface and a reduced sensitivity toward the inhibitory effects of Iloprost, but prostacyclin receptors can be recycled rapidly to the platelet surface in a functionally active form after withdrawal of Iloprost [58]. Exposure of platelets to low doses of Iloprost might not lead to desensitisation and internalisation of platelet prostaglandin receptors. These suggest that Iloprost at low doses could be used to protect stored platelets from apoptosis these platelets still retain their functional activity. Iloprost may have profound implications for the treatment of many common diseases caused by platelet apoptosis. This is the work of the future.

In conclusion, Iloprost concentration‐dependently attenuates platelet function and apoptosis by elevating PKA activity, whereas Iloprost at low concentrations inhibits only platelet apoptosis but not platelet function. Iloprost at low doses, in many common diseases resulting from platelet apoptosis or in stored platelets, may be used for extending platelet life span or elevating peripheral platelet levels by inhibiting platelet apoptosis while having no effect on platelet function, and Iloprost at moderate doses may be used as an antiplatelet drug for preventing the formation of unwanted thrombi.

## Author Contributions

**Xuexiang Wang:** formal analysis (lead), investigation (lead), methodology (lead), writing – original draft (lead), writing – review and editing (equal). **Shuang Chen:** formal analysis (equal), investigation (equal), methodology (equal), writing – review and editing (equal). **Jun Wan:** investigation (equal), methodology (equal), writing – review and editing (equal). **Chunliang Liu:** investigation (equal), methodology (equal), writing – review and editing (equal). **Yan Yan:** formal analysis (equal), investigation (equal), methodology (equal). **Muhammad Shoaib Khan:** formal analysis (equal), investigation (equal), methodology (equal). **Ziyu Zhao:** investigation (equal), methodology (equal). **Kang Sun:** formal analysis (equal), methodology (equal). **Renping Hu:** formal analysis (equal), methodology (equal). **Mengnan Yang:** formal analysis (equal), methodology (equal). **Yue Xia:** formal analysis (equal), methodology (equal). **Kesheng Dai:** conceptualization (lead), funding acquisition (lead), project administration (lead), supervision (lead), writing – review and editing (equal).

## Conflicts of Interest

The authors declare no conflicts of interest.

## Supporting information


Appendix S1.

## Data Availability

Original data could be shared upon reasonable request by contacting the corresponding author.
