# Supplementary material for: Iloprost Concentration‐Dependently Attenuates Platelet Function and Apoptosis by Elevating PKA Activity
Source: J Cell Mol Med. 2025 Feb 10;29(3):e70403. doi: 10.1111/jcmm.70403 (PMC11810529; doi:10.1111/jcmm.70403)
Supplement: Supplementary file 1 — Appendix S1. [file JCMM-29-e70403-s001.docx]

Supplementary data


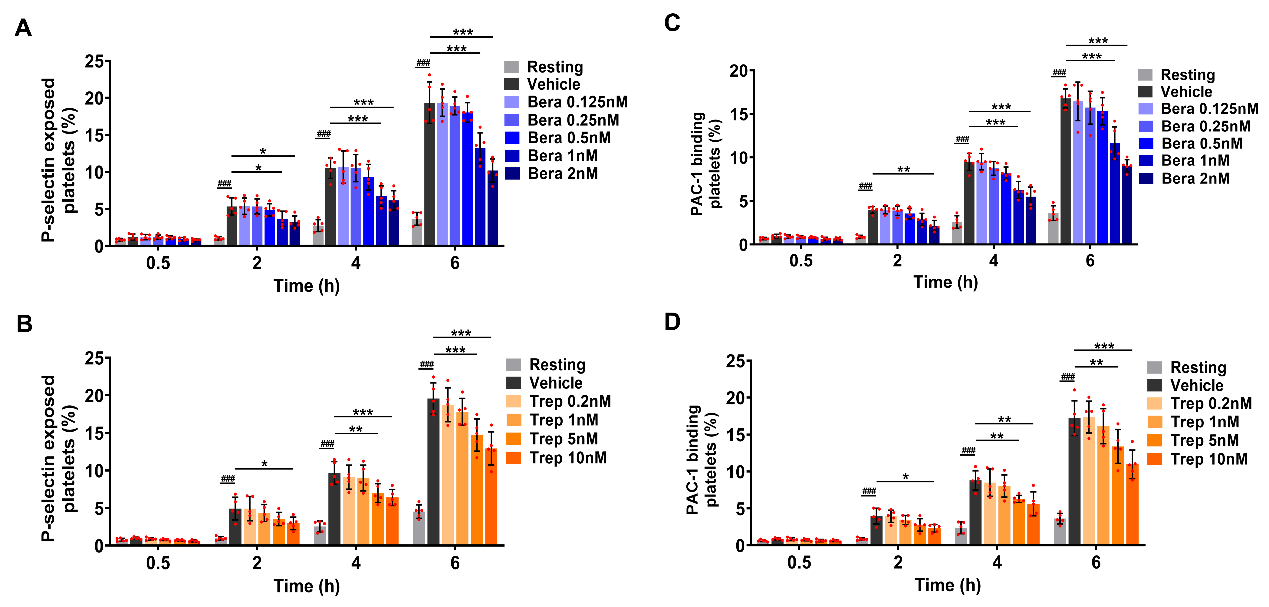


**Figure S1.** **Beraprost (Bera) and** **Treprostinil (Trep) concentration-dependently attenuate platelet activation.** (*A-D*) Washed platelets from healthy human volunteers were pretreated with different concentrations of Bera (0.125, 0.25, 0.5, 1 and 2 nM) or Trep (0.2, 1, 5 and 10 nM) or 0.1% DMSO (vehicle control and resting platelets) at 37 °C for 5 min, followed by activation with 10 μg/mL SZ-2 or mouse IgG (resting platelets) at 37 °C. Time course of platelet activation occurring in human platelets was analyzed by flow cytometry. Beraprost (*A*) and Treprostinil (*B*) inhibited P-selectin exposure (CD62P) during α-granule secretion. (*A* and *B*) Quantitative analysis of platelet P-selectin expression on platelet surface. Beraprost (*C*) and Treprostinil (*D*) inhibited integrin αIIbβ3 activation. (*C* and *D*) Quantitative analysis of platelet αIIbβ3 activation (PAC-1 binding) on platelet surface. Data are expressed as mean ± SD (n=5). ^#^*P* < 0.05, ^##^*P* < 0.01, ^###^*P* < 0.001 compared to resting platelets, **P* < 0.05, ***P* < 0.01, ****P* < 0.001 compared to vehicle control, by two-way ANOVA followed by Dunnett's multiple comparisons test.


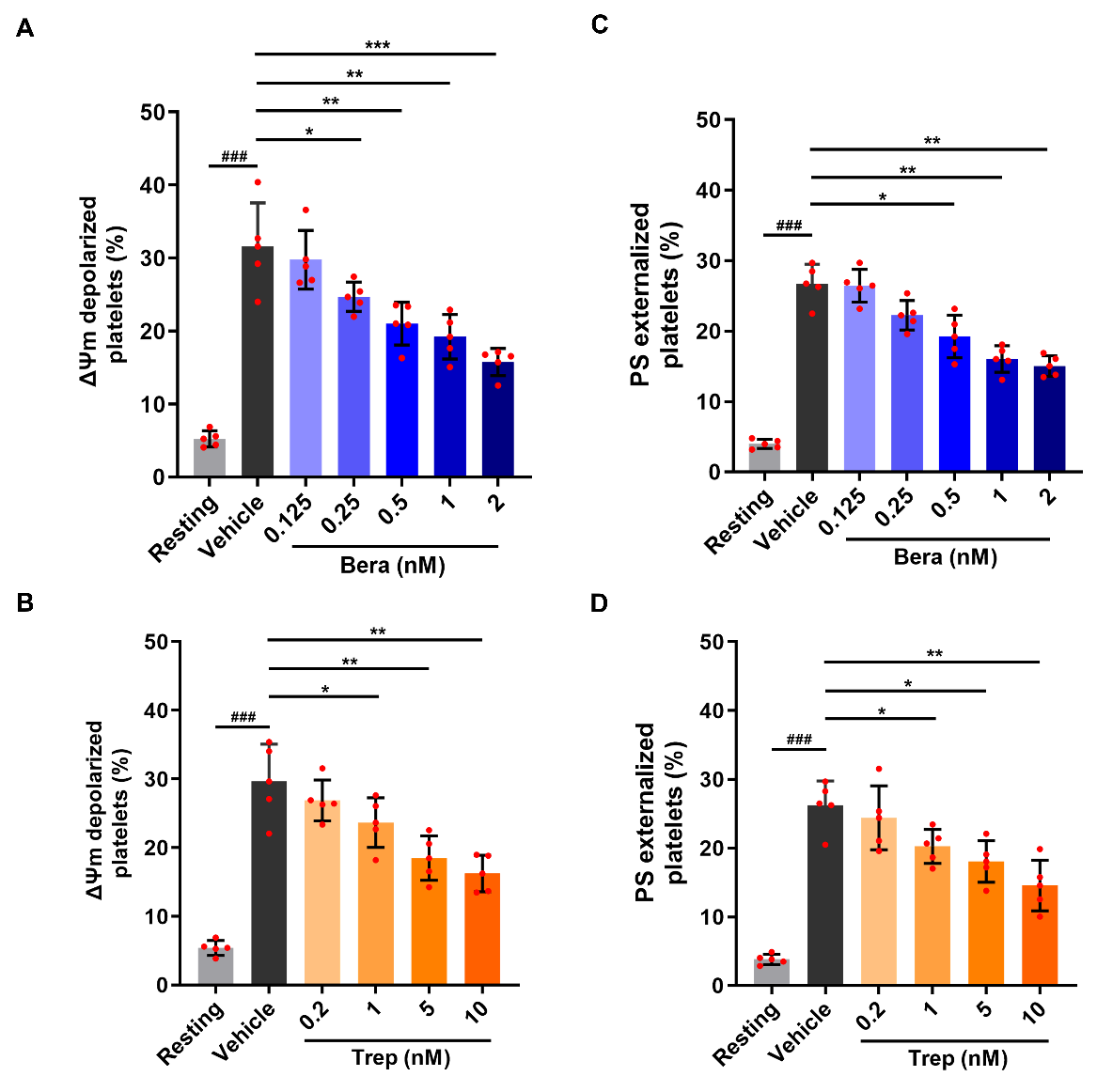


**Figure S2. Beraprost and Treprostinil concentration-dependently inhibit platelet ΔΨm depolarization and PS exposure.** (*A-D*) Washed platelets from healthy human volunteers were pretreated with different concentrations of Bera (0.125, 0.25, 0.5, 1 and 2 nM) or Trep (0.2, 1, 5 and 10 nM) or 0.1% DMSO (vehicle control and resting platelets) at 37 °C for 5 min, followed by activation with 10 μg/mL SZ-2 or mouse IgG (resting platelets) at 37 °C for 6 h. ΔΨm depolarization and PS exposure occurring in human platelets were analyzed by flow cytometry. Beraprost (*A*) and Treprostinil (*B*) inhibited platelet ΔΨm depolarization. (*A* and *B*) Quantitative analysis of platelet ΔΨm depolarization. Beraprost (*C*) and Treprostinil (*D*) inhibited PS exposure. (*C* and *D*) Quantitative analysis of platelet PS exposure. Data are expressed as mean ± SD (n=5). ^#^*P* < 0.05, ^##^*P* < 0.01, ^###^*P* < 0.001 compared to resting platelets, **P* < 0.05, ***P* < 0.01, ****P* < 0.001 compared to vehicle control, by two-way ANOVA followed by Dunnett's multiple comparisons test.
